# FOSTERING GENERATIVITY: MENTORSHIP AND KNOWLEDGE EXCHANGE IN GERONTOLOGY

**DOI:** 10.1093/geroni/igae098.1651

**Published:** 2024-12-31

**Authors:** David Chiriboga

**Affiliations:** University of South Florida, Tampa, Florida, United States

## Abstract

In this presentation, Dr. David Chiriboga will lead a panel discussion sharing his invaluable experiences and insights on mentorship within the field of gerontology. Dr. Chiriboga’s impactful work spans diverse research areas, including studies on health disparities, family-centered interventions for Alzheimer’s disease, and barriers to healthcare among older Korean immigrants. Renowned for his role as a mentor, Dr. Chiriboga has nurtured numerous researchers, shaping the landscape of aging studies. His dedication to fostering talent extends beyond academia, evidenced by his mentorship in initiatives like the American Society on Aging’s New Ventures in Leadership program. Acknowledged for his exceptional mentorship, Dr. Chiriboga has received prestigious awards such as the Hiram J. Friedsam Mentorship Award from the Academy for Gerontology in Higher Education and the Minority Mentorship Award from the Gerontological Society of America (GSA). In his presentation, Dr. Chiriboga will illuminate the significance of mentorship and the culture surrounding it, drawing from his wealth of experience. The panel discussion will further explore mentorship and generativity development in gerontology. Following the moderated discussion, the audience will engage in an interactive question-and-answer session. By emphasizing the importance of mentorship and inter-generational knowledge exchange, this presentation aims to illuminate strategies for retaining the legacy of successful mentoring within the GSA and advancing the field through collaborative learning and development.

